# The *Perilipin* Homologue, *Lipid Storage Droplet 2*, Regulates Sleep Homeostasis and Prevents Learning Impairments Following Sleep Loss

**DOI:** 10.1371/journal.pbio.1000466

**Published:** 2010-08-31

**Authors:** Matthew S. Thimgan, Yasuko Suzuki, Laurent Seugnet, Laura Gottschalk, Paul J. Shaw

**Affiliations:** Department of Anatomy and Neurobiology, Washington University School of Medicine, St. Louis, Missouri, United States of America; Texas A&M University, United States of America

## Abstract

Extended periods of waking result in physiological impairments in humans, rats, and flies. Sleep homeostasis, the increase in sleep observed following sleep loss, is believed to counter the negative effects of prolonged waking by restoring vital biological processes that are degraded during sleep deprivation. Sleep homeostasis, as with other behaviors, is influenced by both genes and environment. We report here that during periods of starvation, flies remain spontaneously awake but, in contrast to sleep deprivation, do not accrue any of the negative consequences of prolonged waking. Specifically, the homeostatic response and learning impairments that are a characteristic of sleep loss are not observed following prolonged waking induced by starvation. Recently, two genes, *brummer* (*bmm*) and *Lipid storage droplet 2* (*Lsd2*), have been shown to modulate the response to starvation. *bmm* mutants have excess fat and are resistant to starvation, whereas *Lsd2* mutants are lean and sensitive to starvation. Thus, we hypothesized that *bmm* and *Lsd2* may play a role in sleep regulation. Indeed, *bmm* mutant flies display a large homeostatic response following sleep deprivation. In contrast, *Lsd2* mutant flies, which phenocopy aspects of starvation as measured by low triglyceride stores, do not exhibit a homeostatic response following sleep loss. Importantly, *Lsd2* mutant flies are not learning impaired after sleep deprivation. These results provide the first genetic evidence, to our knowledge, that lipid metabolism plays an important role in regulating the homeostatic response and can protect against neuronal impairments induced by prolonged waking.

## Abstract 2

Starvation, which is common in the wild, appears to initiate a genetic program that allows fruitflies to remain awake without the sleepiness and cognitive impairments that typically follow sleep deprivation.

## Author Summary

It is well established in humans that sleep deficits lead to adverse outcomes, including cognitive impairments and an increased risk for obesity. Given the relationship between sleep and lipid stores, we hypothesized that metabolic pathways play a role in sleep regulation and contribute to deficits induced by sleep loss. Since starvation has a large impact on metabolic pathways and is an environmental condition that is encountered by animals living in the wild, we examined its effects on sleep in the fruit fly *Drosophila melanogaster*. Interestingly, when flies are starved they display an immediate increase in waking. However, in contrast to sleep deprivation, waking induced by starvation does not result in increased sleepiness or impairments in short-term memory. To identify the mechanisms underlying these processes, we evaluated mutants for genes that have been shown to alter an animal's response to starvation. Interestingly, *brummer* mutants, which are fat, show an exaggerated response to sleep loss. In contrast, mutants for *Lipid storage droplet 2* are lean and are able to stay awake without becoming sleepy or showing signs of cognitive impairment. These results indicate that while sleep loss can alter lipids, lipid enzymes may, in turn, play a role in regulating sleep and influence the response to sleep deprivation.

## Introduction

Insufficient sleep adversely affects both endocrine and metabolic processes, resulting in glucose intolerance, insulin resistance, and obesity [1]. Moreover, sleep deprivation results in extensive physiological impairments in both vertebrates and invertebrates, including, but not limited to, cognitive impairments and death [2]–[6]. Sleep homeostasis is defined as the increase in sleep observed following sleep loss. It is theorized that sleep homeostasis restores vital biological functions degraded during sleep deprivation. Unfortunately, precisely which processes need restoration remains a matter of speculation and debate. A common strategy to identify pathways that regulate sleep homeostasis has been to compare animals that have been sleep deprived with a control group that has been sleeping or has had the opportunity to sleep. While many processes have been identified with this approach [7]–[10], the extent to which they play a role in sleep homeostasis is largely unknown.

An alternative strategy for identifying pathways associated with sleep homeostasis is to take advantage of the observation that environmental conditions influence the response to prolonged waking [11]–[13]. Indeed, many behaviors are influenced by interactions between genes and the environment [6],[14]–[19]. That is, an individual will respond to the specific demands/constraints of their current environment by altering their physiological response in order to optimize their chances of success. Thus a similar challenge that occurs in two distinct environments may differentially activate specific pathways such that contrasting outcomes are observed. For example, at room temperature, flies mutant for the canonical clock gene *cycle* (*cyc^01^*) show an exaggerated sleep rebound (∼10-fold greater than wild-type flies) and begin to die if kept awake for 10 h [6]. However, if *cyc^01^* mutants are exposed to an environment with a higher temperature, they no longer exhibit an exaggerated sleep rebound after sleep deprivation, and they do not die when kept awake for 10 h [6],[20]. Thus, it may be possible to more efficiently identify pathways that protect flies from the negative effects of waking by evaluating the differential responses to sleep loss that occur in two distinct environments.

One such environmental condition that may be particularly useful for contrasting with sleep deprivation is starvation. It has long been recognized that, in several species, the lack of food availability increases the duration of waking [21]–[25]. Moreover, rats respond to chronic total food deprivation with a linear increase in wakefulness, and when allowed access to food, they do not show increases in non-rapid eye movement sleep [26]. Similarly, fasting humans show a reduction in sleep time and increased sleep latency [27]. It has been suggested that animals that are able to remain alert and vigilant in the absence of food might have a selective advantage over animals that accrue sleep debt at a normal rate [24]. Thus, identifying the unique physiological responses to waking induced by starvation as compared to waking induced by sleep deprivation may provide insights into mechanisms underlying sleep homeostasis.

We evaluated the consequences of waking induced by starvation and contrasted them to an equivalent amount of waking induced by sleep deprivation. Our results indicate that while sleep deprivation robustly activates sleep homeostasis and results in learning impairments, these negative consequences are not observed following waking induced by starvation. Although *cyc^01^* flies die from sleep loss in 10 h, they can withstand ∼28 h of waking induced by starvation [6]. We demonstrate that *cyc^01^* mutants have increased triglyceride stores compared to background controls, suggesting that genes involved in lipid metabolism may influence the response to extended waking. Two likely candidates are *brummer* (*bmm*), a homologue of adipose triglyceride lipase, and *Lipid storage droplet 2* (*Lsd2*), a homologue of *Perilipin*. *bmm* mutants exhibit increased triglycerides and resistance to starvation [28], while *Lsd2* mutants are lean and sensitive to starvation [29]. We show here that *bmm* and *Lsd2* mutants play a role in sleep regulation. That is, *bmm* mutants displayed a large homeostatic response following sleep deprivation, while *Lsd2* mutants had a suppressed sleep rebound. Importantly *Lsd2* mutants maintain their ability to learn even in the face of sleep loss, indicating that they are protected from the negative effects of waking. To our knowledge, these results provide the first genetic evidence that lipid metabolism plays a role in regulating sleep homeostasis.

## Results

### Waking Induced by Starvation Does Not Initiate a Sleep Rebound

To begin, we investigated the effects of starvation on sleep homeostasis in flies mutant for *cyc^01^* and *period* (*per^01^*). We chose to evaluate *cyc^01^*mutants first due to their extreme sensitivity to sleep loss.

**Figure 1:**
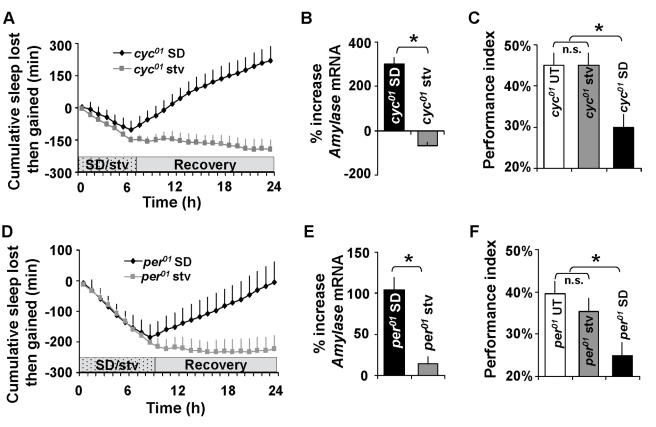
Starvation produces a waking state that does not activate sleep homeostasis. (A) *cyc^01^* flies show a large homeostatic response after 7 h of sleep deprivation (SD, black) but do not display a homeostatic response following waking induced by 7 h of starvation (stv, gray). The sleep deprivation and starvation experiments were conducted in parallel on female siblings whose baseline sleep was monitored for 3 d; experiments were conducted in constant darkness (DD). Cumulative sleep lost or gained during SD or starvation. A negative slope indicates sleep lost, and a positive slope indicates sleep gained; when the slope is zero, recovery is complete. Stippled bar indicates treatment and white bar indicates recovery (*n* = 72 for each group, data are presented as mean ± SEM). (B) *Amylase* mRNA for sleep deprived and starved *cyc^01^* flies expressed as a percentage change from age-matched untreated controls as measured by QPCR (*n* = 3 replicates of 20 heads/group; *p<.05 Student's *t* test). (C) APS performance is significantly disrupted by SD but is unchanged following starvation in *cyc^01^* flies. The performance index is the number of photonegative choices during the last 4 trials of a 16-trial test; a higher score indicates learning. One way ANOVA *F*[_2,30_]  = 5.26; *n* = 10/group; *p<.05 modified Bonferroni Test. (D) *per^01^* flies show a large sleep rebound following 7 h of sleep deprivation but do not display a homeostatic response following 7 h of starvation (*n* = 72 for each group). (E) *Amylase* mRNA for sleep deprived and starved *per^01^*flies expressed as a percentage change from age-matched untreated controls as measured by QPCR (*n* = 3 replicates of 20 heads/group; *p<.05 Student's *t* test). (F) APS performance is significantly disrupted by SD but is unchanged following starvation in *per^01^* flies. One way ANOVA *F*[_2,26_]  = 2.6; *p<.05 modified Bonferroni Test.

That is, in contrast to wild-type flies and other clock mutants, *cyc^01^* flies show an exaggerated homeostatic response after short-term sleep deprivation and, as a group, begin to die if kept awake for 10 h [6]. When *cyc^01^* flies are placed into recording tubes with agar and water (starvation), they exhibit an immediate and sustained increase in waking behavior (Figure 1A, Figure S1); when placed back on to their standard diet 7 h later, sleep simply returned to baseline with no evidence of a sleep rebound (Figure 1A, squares). The effects of starvation were contrasted with an equivalent amount of sleep deprivation induced using the sleep nullifying apparatus (SNAP), an automated sleep deprivation device that has been found to keep flies awake without nonspecifically activating stress response genes [6]. Sleep deprivation, starvation, and control treatments were conducted concurrently in arrhythmic *cyc^01^* siblings maintained in constant darkness (DD). Consistent with our previous results, *cyc^01^*flies that were sleep deprived using the SNAP displayed an exaggerated homeostatic response (Figure 1A, diamonds); locomotor activity levels between sleep deprived and starved siblings were not significantly different (unpublished data). To confirm that sleep deprived flies could eat during the deprivation protocol and were not starved, we assessed food intake by placing flies on food with a blue dye. Following 7 h of sleep deprivation, 13 out of 14 *cyc^01^* flies clearly exhibited evidence of blue dye in their abdomen while 11 out of 13 untreated baseline controls exhibited blue dye as rated by an observer blind to condition. Spectrophotometric data confirmed these results (absorbance at 625 λ: 8.7×10^−3^/fly and 13.5×10^−3^/fly, respectively). Thus, both the untreated and sleep deprived flies have access to and consume food throughout the treatment period. Activity, sleep, and feeding behavior of flies immediately following sleep deprivation and starvation can be seen in Video S1. It is important to note that with longer durations of starvation, flies begin to display sleep homeostasis indicating that the cost of waking does indeed accrue in the absence of food (Figure S2). However, our data indicate that *cyc^01^*flies have a qualitatively different response to ∼7 h of waking induced by starvation versus 7 h of waking induced by sleep deprivation.

The lack of a homeostatic response following starvation may represent either an adaptation that allows animals to better withstand the negative effects of waking, or it may simply reflect a physiological impairment that globally disrupts several regulatory processes, including sleep homeostasis. To distinguish between these two possibilities, we evaluated *Amylase* transcript levels, a known biomarker of sleepiness in flies, to determine if it was elevated following starvation. We have previously shown that, in flies, *Amylase* levels are only elevated following waking conditions that are associated with increased sleep homeostasis and are not induced by stress [30]. As seen in Figure 1B, *cyc^01^* flies that are sleep deprived for 7 h exhibit a large increase in *Amylase* mRNA while *Amylase* mRNA levels remain unchanged in *cyc^01^* siblings starved for 7 h. Thus, both *Amylase* mRNA levels and the absence of a sleep rebound indicate that, although starvation increases waking, it may not increase sleep drive.

Given that starvation is a metabolic challenge, waking induced by starvation could potentially disrupt the normally tight association that is typically observed between *Amylase* and sleepiness [30]. Therefore we utilized a second, independent behavioral assay to evaluate the functional consequences of waking induced by starvation in *cyc^01^* flies. We chose to evaluate learning, since deficits in learning and memory are well-conserved consequences of sleep deprivation [4],[5],[31]. Learning was examined using Aversive Phototaxic Suppression (APS) [32]. In this task, flies are individually placed in a T-maze and allowed to choose between a lighted and darkened chamber. During 16 trials, flies learn to avoid the lighted chamber that is paired with an aversive stimulus (quinine/humidity). The performance index is calculated as the percentage of times the fly chooses the dark vial during the last 4 trials of the 16-trial test [5],[33]. Consistent with our previous results, 7 h of sleep deprivation resulted in a significant reduction in performance (Figure 1C, black). However, no learning deficits were observed following an equivalent amount of waking induced by starvation (Figure 1C, gray). Our previous studies have shown that the mechanical stimulus used to keep the animals awake does not disrupt performance [5]. Importantly, the time required for the fly to complete the 16 trials (TCT) was not modified by either sleep deprivation (15.5±0.55 min) or starvation (14.5±0.76 min) compared to controls (14.4±0.63 min), indicating that differences in performance are unlikely due to alterations in motivation. Moreover, starvation did not alter sensory thresholds (Table S1) as measured by either the Photosensitivity Index (PI; percentage of photopositive choices in 10 trials in the absence of quinine) or the Quinine Sensitivity Index (QSI; time in seconds flies reside on the non-quinine side of a chamber) consistent with our previous results indicating that sleep deprivation does not alter PI or QSI [5]. The magnitude of the learning deficit observed in *cyc^01^* flies following sleep deprivation is similar to that previously reported for sleep-deprived wild-type flies, flies lacking Mushroom Bodies and classic memory mutants [5],[33]. Moreover, the deficits in learning following sleep loss in flies are within the range of effect sizes observed following sleep loss in humans and rodents across a number of cognitive domains [34]–[37]. Thus, in contrast to 7 h of waking induced by sleep deprivation, 7 h of waking induced by starvation does not induce (1) a homeostatic response, (2) an increase in the expression of a biomarker of sleepiness, or (3) learning impairments.

We next tested whether starvation would alter behavior in *per^01^* flies. We chose to evaluate *per^01^* mutants since our previous results suggested that they are more sensitive to the lethal effects of starvation [6]. As seen in Figure 1D starvation resulted in an immediate and sustained increase in waking that was not compensated for by a homeostatic response when flies were placed back on to their normal diet. Similar to the effects observed in *cyc^01^* mutants, *Amylase* levels were not elevated in *per^01^* mutants following waking induced by starvation but were elevated by an equivalent amount of waking induced by sleep deprivation (Figure 1E). Finally, while 7 h of waking induced by sleep deprivation resulted in significant decrements in performance in the APS, 7 h of waking induced by starvation did not result in learning deficits (Figure 1F). Starvation did not alter TCT in *per^01^* mutants compared to untreated controls (14.7±0.86 versus 13±0.38 min, respectively) and did not alter PI or QSI. Thus, two different clock mutants exhibit similar response to sleep deprivation and starvation as measured by sleep homeostasis, *Amylase* and learning.

**Figure 2:**
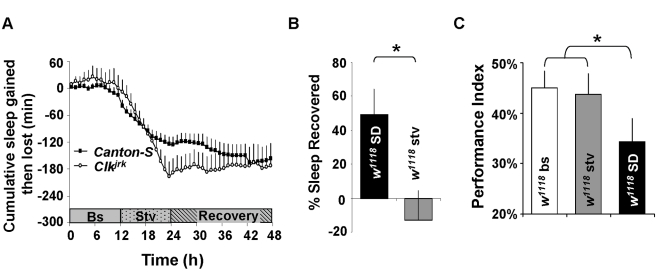
Waking induced by starvation does not activate sleep homeostasis in wild-type flies or mutants for *Clock*. (A) Starvation induces waking in Wild-type *Canton-S* flies (squares, *n* = 16) and flies mutant for the canonical clock gene *Clock* (*Clk^jrk^*, diamonds, *n* = 16). *CS* flies were kept on a 12∶12 LD schedule and starved for 12 h during the dark period (stippled). *Clk^jrk^* mutants were maintained under DD and starved for 12 h. At the end of the starvation period, flies were then placed back on to normal food into LD or DD, respectively, for recovery (black). Cumulative sleep lost or gained during was calculated. Data are presented as mean ± SEM. (B) Recovery sleep measured in *w^1118^* flies following 12 h of sleep deprivation (*n* = 18) or 12 h of starvation (*n* = 9) during the dark period. % sleep recovered is calculated for each individual as a ratio of the minutes of sleep gained above baseline during recovery divided by the total min of sleep lost during sleep deprivation. Sleep homeostasis was larger in sleep deprived flies versus their starved siblings (* *p* = 0.018 by Student's *t* test). (C) Starved *w^1118^* flies (gray bar, *n* = 8) exhibit similar learning scores after extended waking versus untreated circadian matched controls (white bar, *n* = 10) while their sleep deprived siblings (black bar, *n* = 8) display impaired learning; One way ANOVA *F*[_2,23_]  = 2.1; *p<.05 modified Bonferroni Test.

The ability of starvation to increase locomotion in *Drosophila* is well documented [38]–[42], suggesting that its effects are likely to extend beyond clock mutants. However, since the effects of starvation on waking have not been quantified directly, we evaluated waking following starvation in flies maintained on a typical light-dark schedule. Wild-type *Canton-S* (*CS*) flies and flies mutant for *Clock* (*Clk^jrk^*) were exposed to starvation for 12 h during the dark period. As seen in Figure 2, (*CS*) flies and *Clk^jrk^* mutants also displayed an immediate increase in waking in the absence of food that is not compensated by a homeostatic response. In *CS* flies, starvation began at a time of day when feeding is normally low [43] and the amount of waking induced by this duration of starvation is lower than in the clock mutants. Since transgenic lines are frequently generated in a *white* mutant background (*w^1118^*), we further examined the effects of starvation in *w^1118^* mutants as means to assess whether available transgenic tools can be applied to this question. As seen in Figure 2B, 12 h of sleep deprivation in *w^1118^* mutants produces a sleep rebound that is similar to that observed for other wild-type flies [44]–[46]. Importantly, waking induced by 12 h of starvation was not compensated by a sleep rebound in *w^1118^* flies (Figure 2B, gray) even though the amount of sleep lost during starvation did not differ from that induced by sleep deprivation (*p* = .22). Next, we determined whether learning would remain intact in *w^1118^* mutants following waking induced by starvation as was observed in *cyc^01^* and *per^01^* flies. Performance in the APS was evaluated in *w^1118^* mutants following 12 h of sleep deprivation and 12 h of starvation. As seen in Figure 2C, waking induced by sleep deprivation resulted in a significant reduction in learning while siblings that experienced a similar amount of waking induced by starvation performed at baseline levels; the amount of sleep lost did not differ between sleep deprived and starved flies (*p* = .23). Thus, starvation increases waking in wild-type flies and three independent clock mutants, suggesting that the effects of starvation are not limited to a specific genotype or genetic background. Together these data indicate that starvation may be a practical environmental intervention that can be used to identify genes underlying sleep homeostasis.

### *cyc^01^* Flies Have Increased Triglycerides

**Figure 3:**
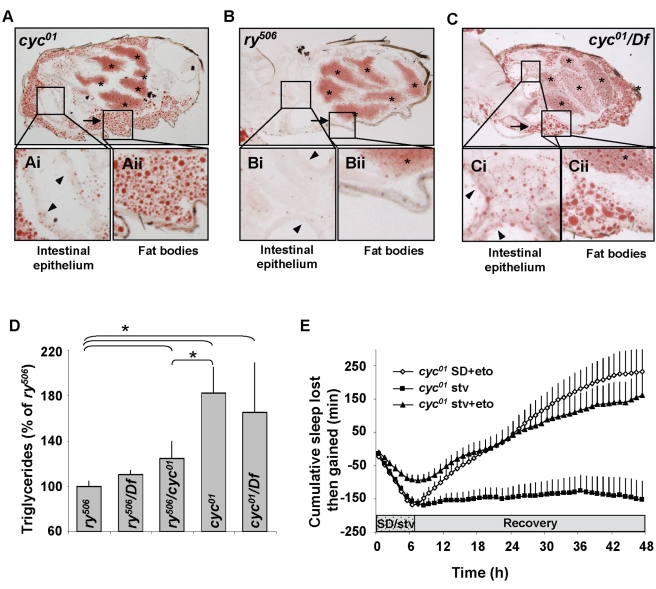
*cyc^01^* flies have increased adiposity. (A) Representative Oil Red O staining, which stains lipids red, reveals high levels of fat stores in the abdomen of female *cyc^01^* flies. Boxed areas (Ai and Aii) are presented at higher magnification. * denotes developing eggs, and arrowheads are placed in the lumen of the gut and point to gut epithelial cells. Arrows point to the location of the fat bodies which abut the cuticle (9 flies were examined). (B) Representative section of *ry^506^* abdomen, the background control for *cyc^01^*. *ry^506^* have little accumulation of lipid droplets in the abdomen in similar areas to (A) as revealed by Oil Red O staining. Boxed areas (Bi and Bii) are presented at higher magnification (9 flies were examined). (C) Representative section of Oil Red O stained *cyc^01^/Df* abdomen reveals high levels of fat stores. (Ci and Cii) are presented at higher magnification (9 flies were examined). (D) Organismal triglyceride (TG) levels are elevated in *cyc^01^*, *cyc^01^*/*Df*, and *cyc^01/^ry^506^*flies compared to *ry^506^* controls; one-way ANOVA for Genotype *F*[_4,38_]  = 3.2; *p<.05 modified Bonferroni Test; *n*≥7 groups/condition, a group was comprised of 10 flies. (E) An elevated homeostatic response is observed in *cyc^01^* mutants that were starved for 7 h (*n* = 45) on 1% agar containing 25 µM etomoxir. No further increase in homeostasis is found in *cyc^01^* sleep deprived for 7 h on food containing 25 µM etomoxir (*n* = 72); compare with Figure 1A. Siblings starved in the absence of etomoxir are shown for comparison (*n* = 92); all experiments were conducted in parallel.

We have previously shown that while *cyc^01^* flies die from sleep loss in 10 h, they can maintain ∼28 h of waking induced by starvation [6]. This result suggested to us the possibility that *cyc^01^* flies might have increased lipid stores. This hypothesis is consistent with recent studies showing that mice mutant for mammalian homologue of *cycle*, *Bmal* ^−*/*−^, show increased total fat content [47]. Thus, we evaluated lipids in *cyc^01^* mutants under baseline conditions. As seen in Figure 3A–Aii, *cyc^01^* mutants had higher levels of lipid stores in their abdomen compared to genetic background controls, *rosy^506^* (*ry^506^*, Figure 3B–Bii). This included increased lipid droplets in gut epithelial cells (Figure 3Ai versus Bi) and the abdominal fat bodies (Figure 3Aii versus Figure 3Bii). Since the *cyc^01^* mutation was generated in a *ry^506^* background (i.e., its full genotype is *cyc^01^, ry^506^*) (see Materials and Methods) [48], *ry^506^* is the appropriate background control for these experiments. To confirm that this phenotype maps to the *cyc* locus, we crossed *cyc^01^* homozygotes with flies carrying the appropriate deficiency *Df(3L)kto2/TM6B, Tb^1^*. The resulting *cyc^01^*/*Df* transheterozygote flies showed an Oil Red O staining pattern that was qualitatively similar to that seen in *cyc^01^* (Figure 3C–Cii). The increased adiposity in *cyc^01^* mutants was confirmed using biochemical measurements of organismal triglyceride (TG) levels [7]. As seen in Figure 3D, both *cyc^01^* and *cyc^01^/Df* show significantly elevated TG levels compared to *ry^506^* controls, whereas the *cyc^01^/ry^506^* heterozygote displayed an intermediate phenotype. Future experiments will be required to determine whether the observed adiposity is due to a direct effect of the *cyc^01^* mutation on lipid stores or whether the elevated TG levels are an indirect consequence of a disrupted circadian clock.

These observations led us to speculate that starvation might protect flies from sleep loss by diverting lipids towards β-oxidation in mitochondria (Figure S3). To test this hypothesis, we reduced long chain free fatty acid (FFA) entry into the mitochondria by feeding *cyc^01^* mutants the carnitine palmitoyltransferase antagonist, etomoxir [49]. Sleep was not modified in control flies fed 25 µM etomoxir for 7 h either during or after administration (unpublished data). Thus, *cyc^01^* flies were fed etomoxir for 7 h during sleep deprivation or 7 h during starvation and then placed back onto their normal food. Etomoxir administration during sleep deprivation did not alter the size of the subsequent homeostatic response (Figure 3E). However, starved *cyc^01^* flies fed etomoxir showed a dramatic increase in sleep homeostasis, which resembled that normally seen after sleep deprivation (Figure 3E). Thus, the mobilization of FFAs may play a role in sleep homeostasis.

### The *Adipose Triglyceride Lipase* Homologue, *Brummer*, Modulates Sleep Homeostasis

**Figure 4:**
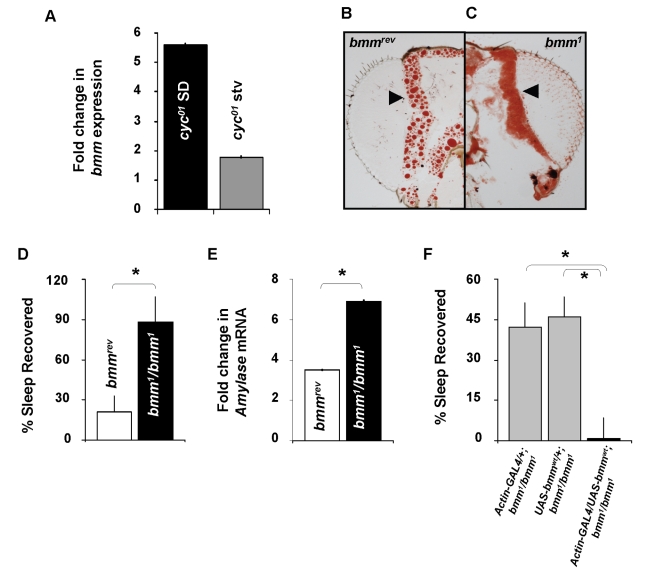
Deletion of *bmm* results in an increased rebound after sleep deprivation. (A) *bmm* mRNA expression is elevated in the heads of *cyc^01^* flies after sleep deprivation but not after starvation. (B) A representative section of *bmm^rev^* head stained with Oil Red O, head fat bodies are denoted by arrowheads; fat droplets are discrete and well defined. (C) A representative section of *bmm^1^* head stained with Oil Red O; head fat bodies are filled with stored lipids without space in between the droplets. (D) *bmm^1^* mutants exhibit a large sleep rebound following 12 h of sleep deprivation compared with genetic background controls, *bmm^rev^*; *n* = 23 and *n* = 21, respectively; * *p* = 0.0033, Student's *t* test. This finding was replicated in 6 independent experiments (*bmm^rev^ n* = 198; *bmm^1^*  = 189). (E) *bmm^1^* mutants have increased *Amylase* mRNA levels after sleep deprivation compared with *bmm^rev^*. Representative results are normalized to untreated siblings (2 replicates of *n* = 20 heads/group). (F) Rescue of sleep homeostasis in *bmm^1^* mutants. A homeostatic response is observed in both parental lines, *Actin-GAL4/+; bmm^1^*/*bmm^1^* (*n* = 88) and *UAS-bmm^wt^*/+;*bmm^1^*/*bmm^1^* (*n* = 105) following 12 h of sleep deprivation. However, the rescue line *Actin-GAL4/UAS-bmm^wt^*; *bmm^1^*/*bmm^1^* (*n* = 55) did not respond to sleep loss with an observable homeostatic response. One way ANOVA *F*[_2,245_]  = 7.00; *p<.001 modified Bonferroni Test.

A *Drosophila* mutant, *bmm^1^*, has recently been described, which has large triglyceride stores and is resistant to starvation due to its inability to efficiently liberate FFA from triglyceride stores. To determine whether transcript levels of *bmm* are responsive to either sleep deprivation or starvation, we evaluated relative changes of *bmm* mRNA from sleep deprived and starved *cyc^01^* mutants. As seen in Figure 4A, sleep deprived *cyc^01^* flies that display a large sleep rebound also exhibit a>5-fold increase in *bmm* transcript levels. However, *bmm* transcripts were increased by <2-fold in *cyc^01^* flies following a similar amount of waking induced by starvation. Together these data suggest that *bmm* may play a role in modulating the response to extended waking. To test this hypothesis, we obtained a deletion mutant, *bmm^1^*, and its genetic background control (*bmm^rev^*). Before evaluating sleep, we wished to confirm that the previously reported lipid phenotype was present in flies maintained under our dietary conditions. Evaluation of organismal TG levels established that *bmm^1^* mutants exhibited increased TG in our hands; 58±6 µg TG/mg fly; *n* = 3 groups of 10 compared to their genetic background controls *bmm^rev^*  = 18±1 µg TG/mg fly; *n* = 4 groups of 10 (*p*<0.001 by Student's *t* test). Although Gronke and colleagues reported increased fat stores in the abdominal fat body, the effects of the *bmm^1^* deletion on the head fat body were not evaluated. As seen in Figure 4B,C, Oil Red O staining of lipids reveals that *bmm^1^* mutants show lipid droplets that are so large that they are hard to discriminate while lipid droplets in the heads of genetic controls (*bmm^rev^*) are small and well defined. Thus *bmm^1^* mutants, like *cyc^01^* mutants, display increased adiposity.

Although *bmm^1^* mutants display increased lipids, their baseline sleep parameters were not severely altered (see Table S2 for baseline sleep characteristics). However, *bmm^1^* mutants had a large sleep rebound following 12 h of sleep deprivation compared to their background controls, *bmm^rev^* (Figure 4D). To determine whether the large sleep rebound in *bmm^1^* mutants was due to an elevated sleep drive, we evaluated *Amylase* mRNA levels following sleep deprivation. As seen in Figure 4E, sleep deprived *bmm^1^* mutant exhibit an elevation in *Amylase* mRNA while their genetic background controls, *bmm^rev^*, showed a less dramatic increase in *Amylase* mRNA. Given that *Amylase* levels are not induced by either paraquat [30] or starvation (Figure 1B) and are not a necessary response to sleep deprivation (see below), the increase in *Amylase* seen in *bmm^1^* is unlikely due to increased sensitivity to stress. Finally, we conducted a rescue experiment to re-introduce wild-type *bmm* (*bmm^wt^*) into an otherwise *bmm^1^* mutant fly. Since lipases are likely to be ubiquitously expressed, we drove *bmm^wt^* using an *Act-GAL4* driver. As seen in Figure 4F, no sleep rebound was observed following 12 h of sleep deprivation in the rescue line (*Act-GAL4/UAS-bmm^wt^; bmm^1^/bmm^1^*) while both parental lines (*Act-GAL4/+, bmm^1^/bmm^1^* and *UAS-bmm/+; bmm^1^/bmm^1^*) displayed a robust homeostatic response (Figure 4F). Together, these data indicate that *bmm* can influence the response to sleep deprivation.

### *Lsd2* Regulates Sleep Homeostasis and Prevents Learning Impairments Following Sleep Loss

**Figure 5:**
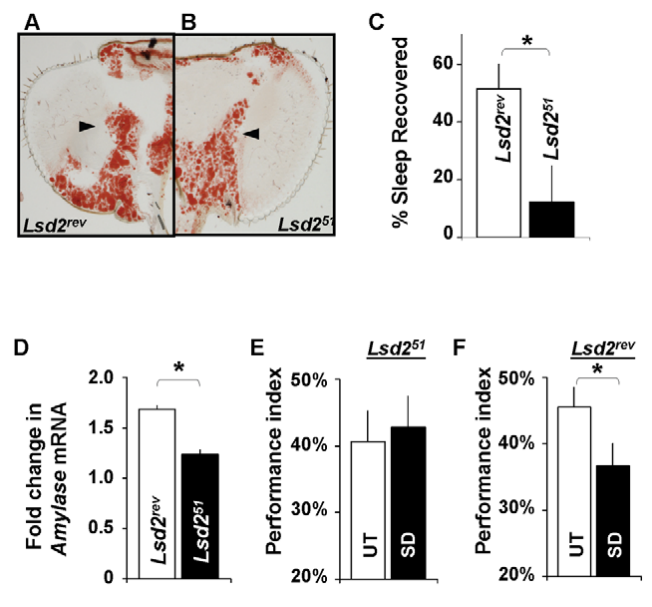
*Lsd2* mutant flies mimic the phenotypes seen in starved *cyc^01^* flies. (A) A representative section of *Lsd2^rev^* head stained with Oil Red O. Head fat bodies are denoted by arrowheads. (B) A representative section of *Lsd2^51^* head stained with Oil Red O. (C) *Lsd2^51^* mutants (*n* = 22) do not exhibit a homeostatic response following 12 h of sleep deprivation while their genetic background control, *Lsd2^rev^* (*n* = 41), do exhibit a sleep rebound following sleep loss. *p = 0.012; Student's *t* test. (D) *Amylase* mRNA levels of *Lsd2^51^* mutants are significantly lower than genetic background controls, *Lsd^rev^*, following 12 h of sleep deprivation. Data are presented as percentage from untreated siblings (2 replicates of *n* = 20 heads/group). *p<.05, Student's *t* test. (E) Learning is not impaired in *Lsd2^51^* mutants (*n* = 7) following 12 h of sleep deprivation compared to untreated controls (*n* = 8); *p* = 0.37 Student's *t* test. (F) Learning is impaired in *Lsd2^rev^* flies (*n* = 15) following 12 h of sleep deprivation compared to untreated controls (*n* = 15).* *p* = 0.036 Student's *t* test.

*Lsd2* is a lipid droplet associated protein with *p*erilipin/*A*DRP/*T*IP47 domain (PAT). PAT proteins regulate lipolysis by either blocking lipase access to droplets and by promoting access when phosphorylated [50]. Thus while *Lsd2* mutants may release and re-esterify fatty acids, they would also be expected to show reduced lipolysis upon stimulation. Interestingly, *Lsd2* mutants (*Lsd2^51^*) display lower levels of TG while *bmm* mutants have higher levels of TG than their respective background controls [29]. This relationship between *Lsd2* and *bmm* makes it of particular interest for further investigation. Indeed, whereas *bmm^1^* mutants are fat and readily survive starvation, *Lsd2^51^* are lean and die rapidly when starved [28],[29]. Together with the observation that loss-of-function mutants for *Lsd2* exhibit phenotypes that share aspects with starvation, we hypothesized that normally fed sleep-deprived *Lsd2* mutants would behave as starved flies and would not compensate for lost sleep with a subsequent sleep rebound. To begin, we confirmed that *Lsd2^51^*mutants were lean (*Lsd2^51^*  = 24±2 µg TG/mg fly; *n* = 4 groups of 10) compared to their genetic controls in which the P-element had been excised (*Lsd2^rev^*  = 34±6 µg TG/mg fly; *n* = 4 groups of 10; *p* = 0.014 by Student's *t* test) [29],[51]. In addition, we evaluated lipids in the head fat body. Although not as dramatic as the change seen in the head of *bmm^1^* mutants, lipid droplets were qualitatively smaller in *Lsd2^51^* mutants compared to genetic background controls, *Lsd2^rev^* (Figure 5A,B). As predicted, *Lsd2^51^* flies did not compensate for lost sleep with a significant increase in sleep over baseline during 48 h recovery from sleep deprivation while genetic controls showed a sleep rebound during this time (Figure 5C). Importantly, *Lsd2^51^* flies did not respond to sleep deprivation with an increase in *Amylase* mRNA levels (Figure 5D), suggesting that they were not sleepy. These data are consistent with our previous results demonstrating that, in flies, *Amylase* levels are responsive to conditions of high sleep drive, do not depend upon the method used to keep the animal awake, and are not simply activated by stress. Thus, *Lsd2^51^* flies showed opposite phenotypes to those seen in *bmm^1^* mutants as measured by lower TG stores, no sleep rebound, and no induction of *Amylase* after sleep deprivation.

As mentioned above, the failure to observe a sleep rebound may simply reflect a physiological impairment that globally disrupts several regulatory processes, including sleep homeostasis. If the genetic lesion associated with *Lsd2^51^* simply disrupts the ability to initiate a homeostatic response, then *Lsd2^51^* flies should be learning impaired following sleep deprivation. However, if disrupting *Lsd2* protects a fly from sleep loss, they should learn following sleep deprivation. A direct test of this hypothesis is achieved within a genotype by determining if performance is reduced following sleep deprivation in comparison with untreated siblings [33]. If a fly is learning impaired following sleep loss, they are considered wild-type. With this in mind, we evaluated learning in *Lsd2^51^* mutants following 12 h of sleep deprivation using APS. As seen in Figure 5E, *Lsd2^51^* mutants maintained normal levels of learning even after being kept awake for 12 h and thus do not display a wild-type response. In contrast, their genetic background controls, *Lsd2^rev^* [28], responded to 12 h of sleep deprivation with a significant reduction in learning (Figure 5F) and are considered to have a wild-type response to sleep loss. It is worth noting that while the performance in *Lsd2^51^* flies appears to be slightly lower than that observed in other lines, including genetic controls, the learning scores are well within the range observed for wild-type flies [5]. Moreover, flies that obtain similar performance levels can achieve lower learning scores following sleep deprivation [33]. Thus *Lsd2^51^* mutants phenocopy starvation as measured by their ability to withstand waking without initiating a homeostatic response or becoming learning impaired.

## Discussion

We have developed a novel strategy to identify pathways involved in sleep homeostasis. This strategy takes advantage of the observation that many behaviors are influenced by interactions between genes and the environment [6],[14]–[19]. We chose to examine starvation because it is common in nature and therefore the response to the absence of food is likely to be evolutionarily conserved. More importantly, starvation is a simple manipulation that can be readily placed under experimental control. We report that starvation induces episodes of waking that are not compensated for by a sleep rebound and do not result in learning deficits. Based upon these results, we then evaluated two genes, *brummer* (*bmm*) and *Lsd2*, which have been shown to modulate the response to starvation [28],[29]. *bmm^1^* mutants, which have increased lipid stores, display an exaggerated sleep rebound. In contrast, mutants for *Lsd2*, which has been reported to mimic some aspects of starvation, are able to withstand the negative effects of waking without compensating for lost sleep or exhibiting the learning deficits that are typically observed after 12 hr of sleep deprivation. These data suggest that proper lipid handling is important for modulating an organism's response to sleep loss. Although the precise mechanisms by which these genes alter sleep regulation remains to be determined, these data represent a first step in the molecular dissection of sleep homeostasis.

It is interesting to note that gene profiling studies in several species have consistently identified genes involved in metabolism as being modified by behavioral state [7],[9],[10],[52],[53]. Indeed, the first gene found to be modified by behavioral state in flies was *fatty acid synthase* [44]. Although many of the specific genes are not identical across studies, it is important to recognize that the categories and pathways are consistent, thereby reinforcing the view that sleep regulatory pathways and lipid metabolism are intimately involved. The impact of sleep deficits on metabolism is now well documented [3],[54]. In humans, sleep deficits are known to result in metabolic disruption and increased adiposity [1],[55]. Similarly, long-term chronic total-sleep deprivation in rodents is also associated with severe metabolic disruption [56]. Thus while our data confirm previous observations that sleep loss activates metabolic genes, we also present data demonstrating that metabolic genes, in turn, can influence sleep regulatory centers as measured by sleep homeostasis. Together these data imply a bi-directional relationship between sleep and metabolism.

It should be noted that lipids are not just a source of energy but are important modulators of cell signaling, gene transcription, metabolism, and appetite [57]. They modify the functional responses of ion channels, synaptic function, and cellular signaling cascades [58],[59]. Lipids also activate G-Protein coupled receptors suggesting that they have an extracellular mode of action [60]. Determining which lipid is able to influence sleep homeostasis is a considerable challenge that cannot be solved using genetic strategies alone. Thus, while our genetic studies have identified important lipid metabolism pathways, additional work will be required to fully elucidate the precise molecular mechanisms that impact the sleep regulatory centers. It is highly likely that future studies will turn to lipidomic analysis. The genes and genetic tools we have identified here may be particularly useful in guiding future lipidomic studies.

We began by contrasting waking induced by sleep deprivation with waking induced by starvation. Interestingly the mutant *Lsd2^51^*, which phenocopies aspects of starvation as measured by low triglyceride stores [28],[29], also phenocopies starvation at the behavioral level. That is, *Lsd2^51^* mutants can withstand 12 h of sleep deprivation without exhibiting any evidence of a compensatory sleep rebound as is seen with starved flies. It is unlikely that mutations in *Lsd2* disrupted the ability of the fly to recover needed sleep since they did not appear sleepy as measured by *Amylase* mRNA. This interpretation is bolstered by the observation that 12 h of waking in *Lsd2^51^* mutants did not result in learning impairments. Learning impairments are a robust consequence of sleep deprivation in mammals and in flies [4],[5],[37]. We have previously shown that neither *Amylase* mRNA levels nor learning impairments can be explained by the method used to keep the animals awake or stress [30]. The observations obtained in both *Lsd2^51^* mutants and starved flies provide additional confirmation of these conclusions. Moreover, these results emphasize the utility in evaluating *Amylase* and learning in addition to sleep homeostasis when interpreting results from genetic studies. Given that sleep homeostasis, *Amylase*, and learning all suggest that *Lsd2* mutants are resilient in the face of sleep loss, understanding the underlying mechanisms may have clinical utility.

At this stage, most of our knowledge about the role of lipid regulation, in general, and Perilipin, in particular, has been derived from mammalian studies, although great strides are being made with *Drosophila* [28],[29],[51],[61]–[63]. The protein product of *Perilipin*, the mammalian homolog of *Lsd2*, surrounds the lipid droplet, thereby preventing access of lipases to the TGs. In addition, Perilipin is able to sequester proteins that activate lipolysis [50]. Mice lacking a functional *Perilipin* gene (*PLIN* ^−*/*−^) display higher levels of basal lipolysis in white adipose tissue (WAT). However, *PLIN* ^−*/*−^ mice do not show the typical increase in lipolysis upon β-adrenergic receptor stimulation [64],[65]. In contrast, mice lacking a functional *Adipose triglyceride lipase* gene (*Atgl* ^−*/*−^), the mammalian homolog of *bmm*, have decreased basal lipolysis. Yet like *PLIN* ^−*/*−^ mice, *Atgl* ^−*/*−^ do not increase lipolysis when stimulated by a β-adrenergic agonist; *Atgl* ^−*/*−^ mice also show reduced lipolysis when stimulated by starvation or cold-stress [66]. These data suggest the possibility that deficits in Perilipin may protect against the negative effects of waking, in part, via a sustained release of FFAs. In any event, future studies will be needed to determine whether the response to sleep deprivation observed in *bmm^1^* and *Lsd2^51^* mutant flies will be observed in *Perilipin* and *Atgl* null mutant mice.

Although the mechanisms underlying sleep homeostasis are largely unknown, adenosine has been implicated as playing a role in both rodents and humans [67]–[69]. Reducing adenosine release from glia or conditionally knocking out the gene *adenosine A1R* in mice (*AdoA_1_R* ^−*/*−^) attenuates the homeostatic response to sleep loss [31],[70]. Interestingly, the attenuated homeostatic response in *AdoA_1_R* ^−*/*−^ mice is associated with learning impairments, further supporting the hypothesis that sleep homeostasis restores vital biological functions degraded during sleep deprivation [31]. The cognitive effects of sleep deprivation may be both task and circuit dependent [31],[70]. Indeed, blocking adenosine release from glia prevents cognitive impairment following sleep loss as measured by novel object recognition [70]. Thus, evaluating cognitive behavior following sleep deprivation provides an important tool for evaluating the functional outcome of a genetic manipulation that alters sleep homeostasis [5]. Together with our data, these results suggest that it is possible to identify genes that can attenuate the negative consequence of waking as defined by both reduced sleep homeostasis and intact cognitive ability following waking.

There are many homologous characteristics of sleep between mammals and flies. In both mammals and flies, sleep and wake states are influenced by monoaminergic neurotransmitters [71]–[74], GABA [75], the immune system, [9],[71],[76], and potassium channel activity, to name but a few. However, the evidence in mammals for a role of lipid metabolism in sleep regulation is limited. The absence of *acyl-coenzyme A dehydrogenase*, an enzyme that participates in β-oxidation, results in the reduction of theta waves during sleep [77]. Pharmacologic blockage of PPARγ results in altered slow wave sleep [78], and fatty acids, such as oleamide and anandamide, that depend on *fatty acid amide hydrolase* for degradation appear to induce sleep alterations [79]. Although a P-element screen in *Drosophila* link metabolic genes to baseline sleep [80], to our knowledge we provide the first demonstration that lipid metabolic enzymes play a role in sleep homeostasis. Given that metabolic pathways are highly conserved between mammals and flies [81] it will be interesting to determine whether lipid metabolism also plays a similar role in mammals.

Diverse species such as the pigeon [11], the white crown sparrow [12], the killer whale [82], the rodent [26], and the fly have each developed adaptations that allow them to minimize the deleterious effects of wakefulness in dangerous or life-threatening situations. These observations emphasize that the environment can have a dramatic impact on how an individual responds to extended waking. Since diverse species have developed these adaptations to events which are common in nature, it is likely that they are under genetic control and provide a selective advantage. That is, in certain circumstances it may be beneficial for an animal to be able to withstand a short period of waking without becoming sleepy or cognitively impaired. Our data showing that homeostasis re-emerges with longer durations of starvation suggest these adaptations will have limits. We fully expect that studies evaluating the adaptations seen in the white crown sparrow and the killer whale will continue to provide additional insights into sleep regulation. However, these model systems are not amenable to genetic dissection. In contrast, starvation is easily applied in the laboratory and can be coupled with genetic model systems such as the fly and the mouse. Thus, one can exploit environmental conditions to provide crucial insights into both the mechanisms of sleep regulation and, perhaps, its function. While this article was in review, another group reported that starvation induces spontaneous waking [87].

## Materials and Methods

### Flies and Husbandry

Flies were reared in standard laboratory conditions, 12∶12 light:dark schedule, standard food (yeast, sucrose, corn syrup, molasses, and agar), 25°C, and 50% humidity. The *cycle^01^* (*cyc^01^*) and *period^01^* (*per^01^*) mutant flies were obtained from Dr. Jeff Hall [48]. This mutation was originally generated in a *ry^506^* background (i.e., the full genotype would be +;+;*cyc^01^, ry^506^*), thus *ry^506^* was used as its background control [48]. *Actin-GAL4*/*CyO* (*Act-GAL4*), *rosy^506^* (*ry^506^*), and *Df(3L)kto2/TMB,Tb^1^* were obtained from the Bloomington Stock Center (Bloomington, Indiana). The null mutation for *brummer* (*bmm^1^*) and the background control (*bmm^rev^*) as well as the *Lsd2* mutant (*Lsd2^51^*) and its background control (*Lsd^rev^*) were obtained as a generous gift from Dr. Ronald Kuhnlein.

### Sleep Recording

Three-day-old flies were placed into 65 mm glass tubes containing standard lab food and monitored with the Trikinetics activity-monitoring system (Waltham, MA) as previously described [6],[44]. Briefly, activity was recorded in 1 min bins and episodes of quiescence ≤5 min were considered sleep. Total sleep time, sleep architecture, and sleep homeostasis were calculated using an in-house program according to criteria previously established [6],[44],[83].

### Sleep Deprivation

Flies were sleep deprived using the sleep-nullifying apparatus (SNAP), which asymmetrically tilted −60° to +60° such that the sleeping flies were displaced during the downward movement 6 times/minute [6],[44]. Flies were deprived of sleep for 12 h between ZT12 (lights out) to ZT0 (lights on) at which point flies were released into recovery where they remained unperturbed for 48 h. The clock mutants *cyc^01^* and *per^01^* were maintained and sleep deprived under constant darkness; sleep deprivation occurred for 7 h during the day between CT0 and CT12. Sleep homeostasis was calculated for each individual as a ratio of the minutes of sleep gained above baseline during recovery divided by the total minutes of sleep lost during sleep deprivation (min gained/min lost). Cumulative difference plots were calculated for each individual fly first by subtracting the minutes of sleep during deprivation and recovery from the corresponding baseline value and summing the difference score with the preceding hour. A negative slope indicates that sleep is being lost; a positive slope indicates sleep gained; and a slope of zero indicates that recovery is complete.

### Starvation and Etomoxir

Starvation is operationally defined as a condition in which the animal has no access to food and during which energy intake drops below levels that the animals would normally experience at that time. Flies were placed into Trikinetics tubes containing a 1% agar solution and then switched back to their normal food at the end of the starvation period. For all genotypes, starvation was carried out in constant darkness at the same time, for the same duration, and under the same conditions as for sleep deprived flies. Durations for starvation and sleep deprivation were based on Figure S2 and [6]. For the clock mutants, *cyc^01^* and *per^01^*, a 7 h treatment was chosen because it maximized the difference in behavioral responses to sleep deprivation and starvation but did not result in lethality. *CS* flies were housed under DD for 3 d. On day 4, starvation was carried out for 12 h during the primary sleep period. The primary sleep period was identified from the previous days' data based upon the average time that the *CS* flies initiated their longest sleep bout. *w^1118^* experiments were carried out under LD conditions. Flies were transferred to starvation media prior to lights out, where they remained for the 12 h dark period. The following morning, flies were placed back on to normal food to evaluate sleep homeostasis or their performance was evaluated in the APS. For etomoxir experiments, flies were placed into tubes containing either 1% agar or standard laboratory food with a final concentration of 25 µm etomoxir. At the end of the manipulation, flies were placed back on to standard laboratory food without etomoxir.

### Learning Assay

The learning paradigm requires flies to inhibit a prepotent attraction towards light and has been previously described [32]. Both dark and lighted vials are covered with filter paper. The filter paper in the lighted vial is wetted with 320 µl of a 10^−1^ M Quinine hydrochloride solution (Sigma, St. Louis, MO). After entering the dark or lighted vial, the choice is recorded and the fly is quickly removed from the vial and placed back at the entrance of the maze. The number of times the fly enters the dark vial is tabulated during 4 blocks of 4 trials. During the test, the light and quinine/humidity appear equally on both the right and left. For an experiment, learning was evaluated by the same experimenter who was blind to genotype and condition. Unless otherwise stated, all flies were tested in the morning between ZT0 and ZT4. For sleep deprived and starved flies, they remained in their respective conditions until tested. Learning scores are normally distributed [5]. Thus, statistical analyses were performed using Systat (Systat, Chicago, IL). Differences were assessed using either a Student's *t* test or analyses of variance (ANOVAs), which were followed by a modified Bonferroni test; unless stated otherwise, all experiments are *n*≥7.

### Photosensitivity

Photosensitivity was evaluated in the T-maze over 10 trials in the absence of filter paper. The lightened and darkened chambers appeared equally on both the left and right. PI is the average of the scores obtained for 5–6 flies ± SEM.

### Quinine/Humidity Sensitivity

Five flies were individually placed at the bottom of a 14 cm cylindrical tube (Becton-Dickson, Franklin Lakes, NJ), which was uniformly lighted. Each half of the apparatus contained separate pieces of filter paper, which could be wetted with quinine or kept dry. The QSI was determined by calculating the time that the fly spent on the dry side of the tube when the other side had been wetted with quinine, during a 5 min period.

### QPCR

Total RNA was isolated from 20 fly heads with Trizol (Invitrogen, Carlsbad, CA) and DNAse I digested. In the case of whole flies, 3–5 flies were frozen and homogenized. cDNA synthesis was performed in triplicate using Superscript III (Invitrogen, Carlsbad, CA), according to manufacturer protocol. In order to evaluate the efficiency of each reverse transcription, equal amounts of cDNA were used as a starting material to amplify *RP49* as previously described [6]. cDNA from comparable reverse transcription reactions were pooled and used as a starting material to run three QPCR replicates. Expression values for *RP49* were used to normalize results between groups. For flies maintained on an LD schedule, both experimental and untreated controls were collected at the exact same circadian time ZT0-1. For clock mutants, the control, sleep deprivation, and starvation experiments were run in parallel and the flies were collected at the same time.

### Triglyceride Measurements

For each genotype, 10 female flies were frozen and stored at −80°C. Lipid measurements were carried out at the Clinical Nutrition Research Unit at Washington University. Flies were weighed and homogenized in a 2∶1 (methanol:chloroform) solution to extract the lipids [84]. The MeOH:chloroform is evaporated using the speed vac, and the lipids were re-suspended in the starting reagent for Infinity (ThermoElectron, Waltham, MA) triglyceride reagent and triglyceride levels detected using the colorometric detection according to the manufacturer's specifications. Lipid levels are quantified using a standard curve of known triglyceride run in parallel.

### Oil Red O Staining

Flies were immobilized with CO_2_, submerged in Optimal Cutting Temperature (Tissue-Tek, Torrance, CA) and frozen on dry ice. 12–15 µm frozen sections were collected on Histobond slides (VWR, West Chester, PA). Sections were fixed in 4% paraformaldhyde in phosophate buffered saline (PBS) and subsequently rinsed in PBS. Slides were then rinsed in 60% isopropanol for 5 min. The solution was changed to Oil Red O stain (Sigma, St. Louis, MO) in 60% isopropanol. Sections were stained for 5–10 min. Slides were rinsed several times in distilled water to get rid of excess stain. Slides were dried and mounted using Glycergel (Wako, Carpinteria, CA). Brightfield Images were taken on a Nikon Eclipse 80i microscope (Belmont, CA) using a Micropublisher 5.0 RTV camera (Q imaging, Surrey, British Columbia, Canada) and visualized with the software package Metamorph (Universal Imaging, Downingtown, PA). Images were optimized for visualization and publication using Adobe Photoshop (Adobe, San Jose, CA).

### Feeding Assay

Flies were transferred onto normal food with 1% (v/v) blue food dye (F D & C Blue Dye no. 1, Durkee). At the end of the measurement period, flies were anesthetized by CO_2_ and the appearance of blue dye in the abdomen through the cuticle was evaluated by an observer blinded to conditions using a Vista Vision dissecting microscope (VWR, West Chester, PA).

Spectrophotometric measurement of feeding was based on [43],[85],[86]. Flies from the visual confirmation of blue dye were frozen on dry ice. Heads were then removed to prevent eye pigment from interfering with the absorbance spectrum of the dye. Fly bodies were homogenized in 200 µL PBS buffer and centrifuged (13,000 rpm) for 25 min. The supernatants were transferred to a new tube, again centrifuged at 13,000 rpm for 25 min, and absorbance was measured at 625 nm. Absorbance per fly was determined by taking the total absorbance from the group and dividing it by the total number of flies, then subtracting the absorbance per fly from control flies fed non-dyed normal food to give a final absorbance reading per fly.

## Supporting Information

Figure S1 **Flies respond rapidly to starvation.** (A) Although *cyc^01^* flies sleep normally during the 30 min prior to starvation, once starvation begins no *cyc^01^* fly is quiescent for ≥5 min (paired *t* test, *p* = 4.91×10−9, *n* = 31). Inset: % of flies sleeping during 30 min of baseline and 30 min of starvation. (B) Counts/waking minute are significantly elevated in *cyc^01^* flies during the first 30 min of starvation compared to waking activity in the preceding 30 min (paired *t* test, *p* = 6.36×10−8, *n* = 31). Inset: % of flies that display an increase in counts/waking minute compared to baseline.
(0.46 MB TIF)

Figure S2 **Starvation reduces the cost of waking for a short time.** Sleep homeostasis was assessed after 7, 14, and 21 h of extended waking induced by starvation in *cyc^01^* flies. At time 0, flies were moved from standard laboratory food to agar and water. After the designated starvation period, flies were placed back on standard food for recovery. For cumulative sleep lost then gained plot, a negative slope indicates sleep lost, a positive slope indicates sleep gained; when the slope is zero, recovery is complete. After 7 and 14 h of starvation, no rebound was observed. In contrast, a rebound was observed after 21 h of waking induced by starvation.
(0.51 MB TIF)

Figure S3 **Model of fatty acid distribution.** Fatty acids must be “activated” to their coenzyme A (CoA) derivatives by Acyl CoA Synthetases (ACS) before they can participate in a wide variety of metabolic pathways. ACSs differ in their chain specificity, subcellular localization, and their tissue distribution. The heterogeneity seen amongst ACSs indicate that they can divert fatty acids into separate biological pathways, including, for example, β-oxidation, membrane synthesis, formation of complex lipids, activation of signaling pathways (e.g., Protein Kinase C), and regulation of gene expression. We hypothesize that these latter roles interact with homeostatic mechanisms and that during starvation, fatty acids are shunted into β-oxidation pathways, minimizing their impact on sleep homeostasis.
(0.50 MB TIF)

Table S1 **Control metrics for the APS.** Control metrics consisting of: Phototaxis index (PI) and Quinine Sensitivity Index (QSI) for each experiment are within a previously observed range of scores that sustain normal learning [5],[33]. No statistically significant differences were found for QSI and PI. Since we have previously shown that neither PI nor QSI are modified by sleep deprivation [5] and that even large changes to PI do not impede the identification of learning impairments following sleep deprivation [33], we only show PI and QSI for starved flies, not sleep deprived flies. The failure to observe changes in TCT in learning-impaired sleep deprived *cyc^01^* flies and starved *cyc^01^* flies that learn is consistent with our previous report that TCT provides no explanatory value for predicting the final performance index, and thus we do not report TCT for other genotypes. ^a^ values are shown in [5].
(0.02 MB XLS)

Table S2 **Sleep parameters for flies used for sleep homeostasis.** Baseline sleep characteristics. Total sleep time (TST), daytime sleep (DTS), nighttime sleep (NTS), latency to the first sleep bout after lights off (latency), average sleep bout duration at night (NBD), and the number of flies evaluated for each genotype (n). All data are presented in minutes and represent mean ± SEM. With the exception of the values in bold-type font, sleep parameters fall well within the normal variability found in wild-type populations of flies [83]. The sleep characteristics shown are intended to be descriptive and thus to simplify the presentation of these data; no statistical comparisons are presented.
(0.02 MB XLS)

Video S1 **Fly behavior after waking induced by starvation and sleep deprivation.** Three minutes of behavior for flies after 7 h of starvation (left, *n* = 4), 7 h of sleep deprivation (right, *n* = 4), or untreated controls (middle, *n* = 4). *cyc^01^* siblings were maintained in constant darkness prior to recording, which subsequently took place in the light. Flies were removed from their respective conditions, placed into Trikinetics tubes containing blue dye, allowed to acclimate for a few minutes, and were then recorded for 3 min. The video has been compressed to conserve space; however, the location of the fly in proximity to the food can be assessed. Given the transfer of flies to new tubes, the amount of sleep is relatively low. The flies depicted are a subset of flies that were used to quantify food intake for the first hour immediately after sleep deprivation and starvation. After an hour on blue dye, 13/13 flies that had been sleep deprived showed blue dye in their abdomen compared to 14/14 untreated controls. Spectrophotometric data confirmed these results (absorbance at 625 λ: 8.57×10−3/fly and 11.57×10−3/fly, respectively). Moreover, flies that were starved for 7 h and placed onto food containing blue dye displayed results similar to that seen in untreated controls (14/14 with blue dye and absorbance at 625 λ: 12.89×10−3/fly). Given the similarities in feeding behavior, it is unlikely that food intake post-treatment is responsible for ameliorating the negative effects of waking.
(10.44 MB MOV)

## Abbreviations

ACS: Acyl CoA Synthetase
Act: Actin
APS: Aversive Phototaxis Suppression
Atgl: Adipose triglyceride lipase
bmm: brummer
cDNA: complementary DNA
Clk: Clock
CS: Canton-S
cyc: cycle
DD: constant darkness
FFA: free fatty acid
LD: Light-Dark schedule
*Lsd2*: Lipid storage droplet 2
PAT: *p*erilipin/*A*DRP/*T*IP47
PBS: phosphate buffered saline
per: period
PI: Photosensitivity Index
*PLIN*: *Perilipin*
qPCR: quantitative reverse transcriptase polymerase chain reaction
QSI: Quinine Sensitivity Index
ry: rosy
SD: sleep deprived
SNAP: sleep nullifying apparatus
stv: starved
TCT: Time to Complete Trial
TG: triglyceride
UT: untreated
w: white
WAT: white adipose tissue
