# Supplementary figures and images for: The Perilipin Homologue, Lipid Storage Droplet 2, Regulates Sleep Homeostasis and Prevents Learning Impairments Following Sleep Loss
Source: PLoS Biol. 2010 Aug 31;8(8):e1000466. doi: 10.1371/journal.pbio.1000466 (PMC2930866; doi:10.1371/journal.pbio.1000466)

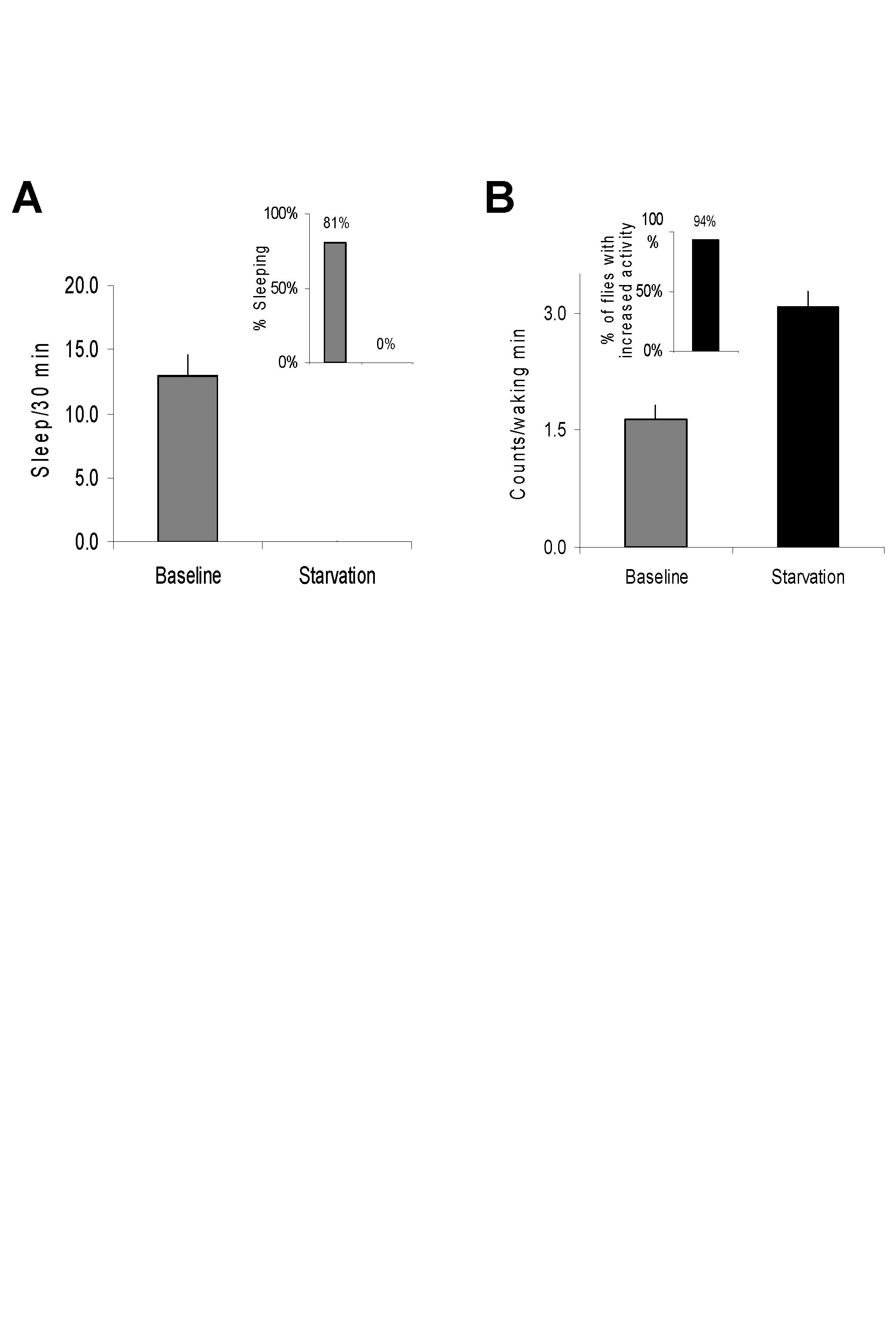

Supplement: Figure S1 — Flies respond rapidly to starvation. (A) Although cyc01 flies sleep normally during the 30 min prior to starvation, once starvation begins no cyc01 fly is quiescent for ≥5 min (paired t test, p = 4.91×10^−9^, n = 31). Inset: % of flies sleeping during 30 min of baseline and 30 min of starvation. (B) Counts/waking minute are significantly elevated in cyc01 flies during the first 30 min of starvation compared to waking activity in the preceding 30 min (paired t test, p = 6.36×10^−8^, n = 31). Inset: % of flies that display an increase in counts/waking minute compared to baseline. (0.46 MB TIF) [file pbio.1000466.s001.tif]

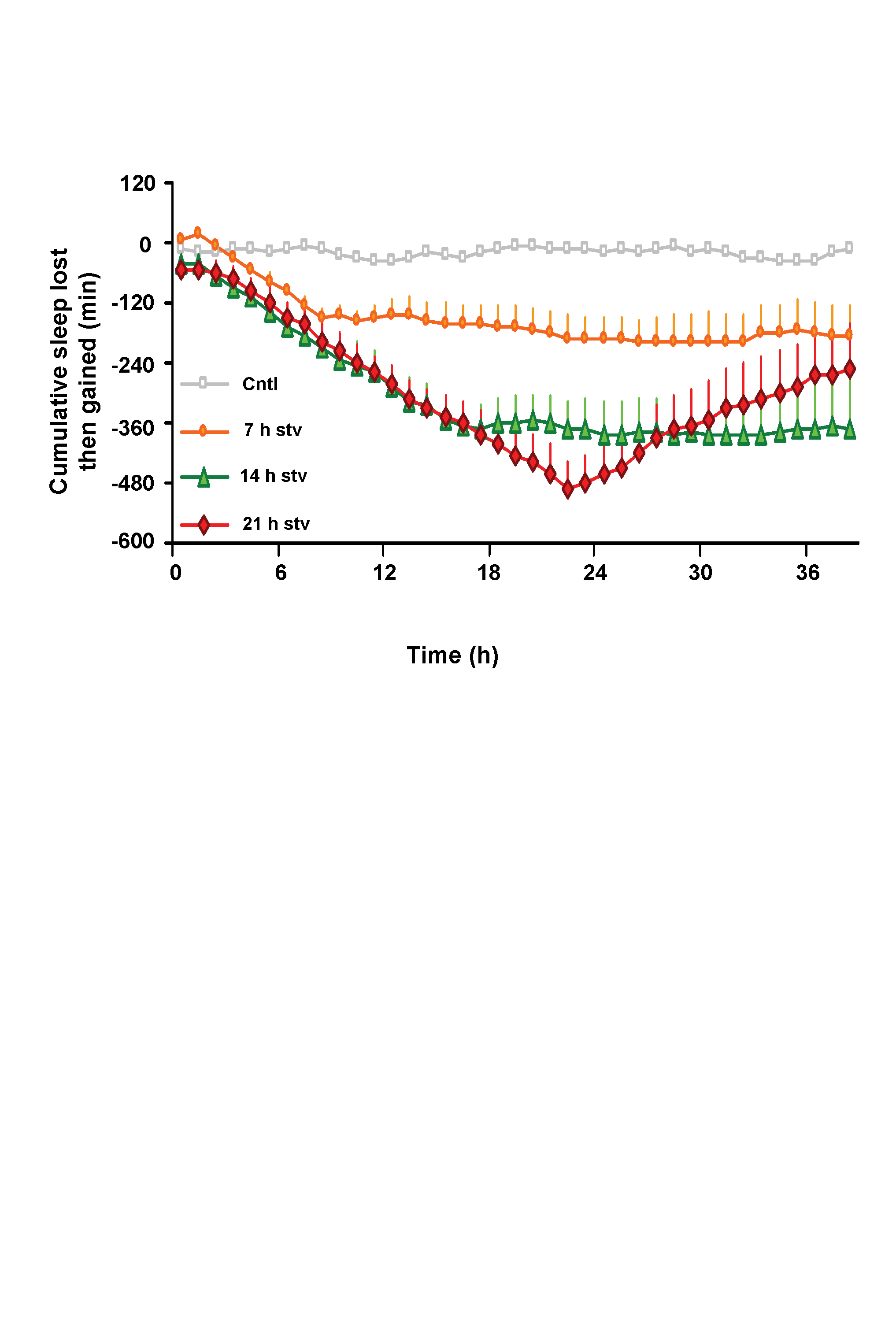

Supplement: Figure S2 — Starvation reduces the cost of waking for a short time. Sleep homeostasis was assessed after 7, 14, and 21 h of extended waking induced by starvation in cyc01 flies. At time 0, flies were moved from standard laboratory food to agar and water. After the designated starvation period, flies were placed back on standard food for recovery. For cumulative sleep lost then gained plot, a negative slope indicates sleep lost, a positive slope indicates sleep gained; when the slope is zero, recovery is complete. After 7 and 14 h of starvation, no rebound was observed. In contrast, a rebound was observed after 21 h of waking induced by starvation. (0.51 MB TIF) [file pbio.1000466.s002.tif]

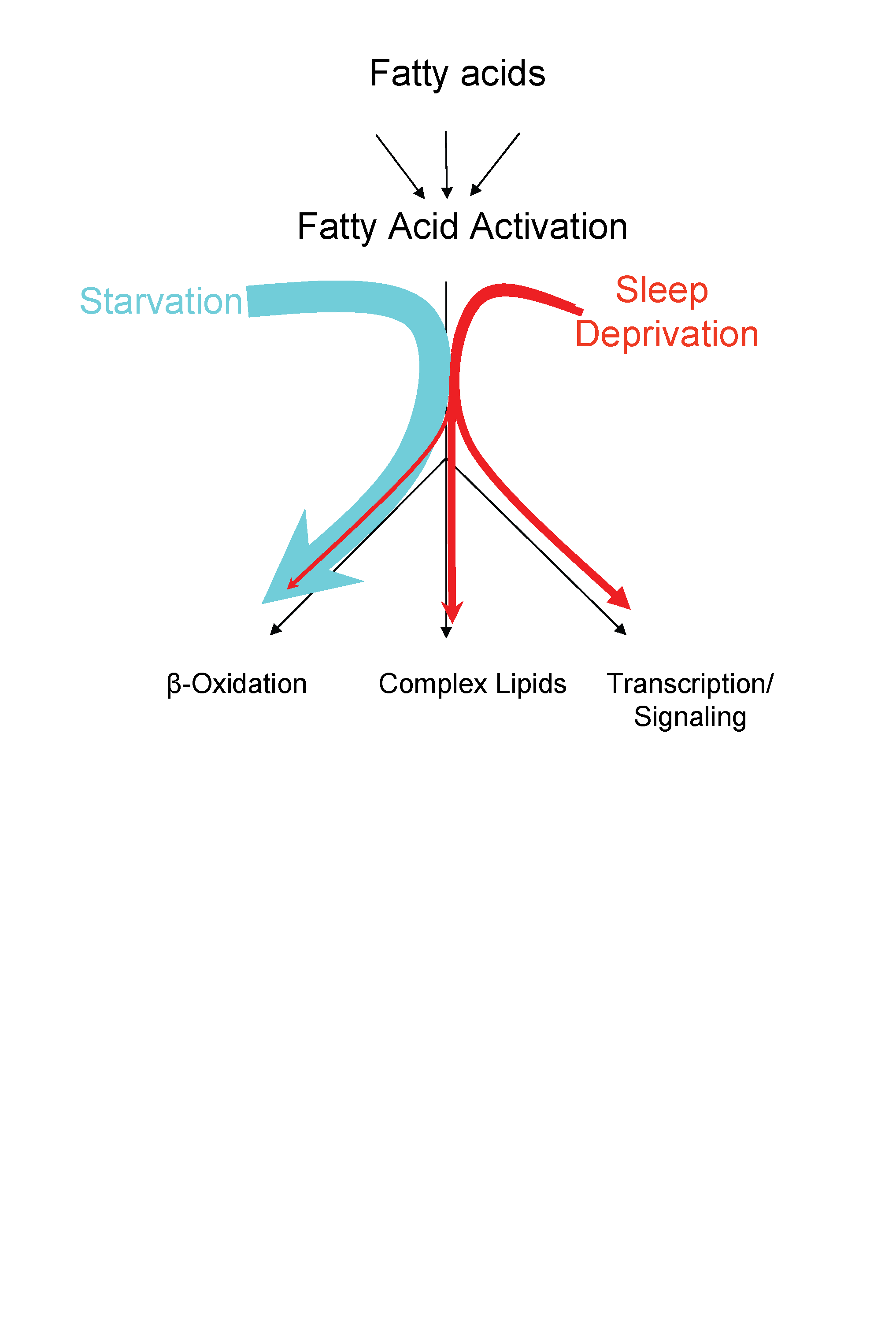

Supplement: Figure S3 — Model of fatty acid distribution. Fatty acids must be “activated” to their coenzyme A (CoA) derivatives by Acyl CoA Synthetases (ACS) before they can participate in a wide variety of metabolic pathways. ACSs differ in their chain specificity, subcellular localization, and their tissue distribution. The heterogeneity seen amongst ACSs indicate that they can divert fatty acids into separate biological pathways, including, for example, β-oxidation, membrane synthesis, formation of complex lipids, activation of signaling pathways (e.g., Protein Kinase C), and regulation of gene expression. We hypothesize that these latter roles interact with homeostatic mechanisms and that during starvation, fatty acids are shunted into β-oxidation pathways, minimizing their impact on sleep homeostasis. (0.50 MB TIF) [file pbio.1000466.s003.tif]
